# Regression from prediabetes to normoglycaemia and the role of cardiometabolic risk factors on the subsequent risk of developing type 2 diabetes

**DOI:** 10.1007/s00125-025-06555-8

**Published:** 2025-10-18

**Authors:** Najmeh Davoodian, Mojtaba Lotfaliany, Rachel R. Huxley, Crystal Man Ying Lee, Julie A. Pasco, Robert J. Adams, Fereidoun Azizi, Alain G. Bertoni, Stephen Colagiuri, Edward W. Gregg, Tiffany K. Gill, Farzad Hadaegh, Davood Khalili, Dianna J. Magliano, Morgana Mongraw-Chaffin, Gita D. Mishra, Jonathan E. Shaw, Masaru Sakurai, Hiroshi Yatsuya, Mohammadreza Mohebbi

**Affiliations:** 1 IMPACT—the Institute for Mental and Physical Health and Clinical Translation, School of Medicine-Barwon Health, Faculty of Health, Deakin University, Geelong, VIC Australia; 2 Biostatistics Unit, Faculty of Health, Deakin University, Geelong, VIC Australia; 3 The George Institute for Global Health, University of New South Wales, Sydney, Australia; 4 Faculty of Health, Deakin University, Melbourne, Australia; 5 School of Population Health, Curtin University, Perth, Australia; 6 Department of Medicine, Western Health, The University of Melbourne, St Albans, Victoria Australia; 7 School of Public Health and Preventive Medicine, Monash University, Melbourne, Victoria Australia; 8 Flinders Health and Medical Research Institute – Sleep Health, Flinders University, Bedford Park, South Australia Australia; 9 Respiratory and Sleep Services, Southern Adelaide Local Health Network, Adelaide, South Australia Australia; 10 Endocrine Research Centre, Research Institute for Endocrine Sciences, Shahid Beheshti University of Medical Sciences, Tehran, Iran; 11 Department of Epidemiology and Prevention, Division of Public Health Sciences, Wake Forest University School of Medicine, Winston-Salem, USA; 12 Faculty of Medicine and Health, The University of Sydney, Sydney, Australia; 13 Charles Perkins Centre, The University of Sydney, Sydney, Australia; 14 School of Population Health, RCSI University of Medicine and Health Sciences, Dublin, Ireland; 15 Department of Epidemiology and Biostatistics, School of Public Health, Imperial College London, London, UK; 16 Adelaide Medical School, University of Adelaide, Adelaide, South Australia Australia; 17 Prevention of Metabolic Disorders Research Centre, Research Institute for Endocrine Sciences, Shahid Beheshti University of Medical Sciences, Tehran, Iran; 18 Department of Diabetes and Population Health, Baker Heart and Diabetes Institute, Melbourne, VIC Australia; 19 Population Health Research, MedStar Health Research Institute, Columbia, MD USA; 20 Australian Women and Girls’ Health Research Centre, School of Public Health, The University of Queensland, Brisbane, Queensland Australia; 21 Department of Social and Environmental Medicine, Kanazawa Medical University, Uchinada, Japan; 22 Department of Public Health and Health Systems, Nagoya University Graduate School of Medicine, Nagoya, Japan

**Keywords:** Cardiometabolic risk factors, Prediabetes regression, Type 2 diabetes

## Abstract

**Aims/hypothesis:**

We aimed to investigate the association between reversion to normoglycaemia among individuals with prediabetes (fasting plasma glucose 5.6–6.9 mmol/l in the absence of other criteria for type 2 diabetes) and the subsequent risk of type 2 diabetes, and to examine whether concurrent favourable cardiometabolic risk factor profiles modify this association.

**Methods:**

We used individual-level data from prospective cohorts in the USA, Australia and Asia. Participants with prediabetes at baseline, with at least two follow-ups (*n*=8191) at median intervals of 2.9 years (IQR 2.3–9.0) and 3.1 years (IQR 2.6–3.6), were classified into restoration of normoglycaemia and persistent prediabetes groups based on the glucose status at the first follow-up (normoglycaemia or prediabetes). Type 2 diabetes occurrence was assessed at subsequent follow-ups. Hierarchical mixed-effects proportional hazards Weibull models estimated type 2 diabetes risk, adjusting for age, sex and cardiometabolic risk factors. A subgroup analysis evaluated the combined association of normoglycaemia restoration and normal cardiometabolic risk factor levels on subsequent type 2 diabetes risk.

**Results:**

In individuals with prediabetes, normoglycaemia restoration compared with persistent prediabetes was associated with a 51% lower risk of developing type 2 diabetes. Even lower risks were observed among individuals with concurrent favourable cardiometabolic profiles, including non-smokers (HR 0.20, 95% CI 0.10, 0.31), and those with normal BMI (0.16, 95% CI 0.06, 0.27), waist circumference (0.22, 95% CI 0.12, 0.33), waist-to-height ratio (0.15, 95% CI 0.03, 0.26), WHR (0.17, 95% CI 0.05, 0.28), and systolic (0.20, 95% CI 0.11, 0.30) and diastolic (0.25, 95% CI 0.12, 0.38) blood pressure, triacylglycerol (0.24, 95% CI 0.13, 0.35) and HDL-cholesterol levels (0.21, 95% CI 0.13, 0.29). Weight loss combined with normoglycaemia restoration was also associated with lower type 2 diabetes risk (0.18, 95% CI 0.07, 0.30) compared with weight gain and persistent prediabetes.

**Conclusions/interpretation:**

Our results suggest that prioritising normoglycaemia restoration during the prediabetes stage in clinical guidelines may contribute to reducing the risk of type 2 diabetes, particularly when accompanied by favourable cardiometabolic health profiles.

**Graphical Abstract:**

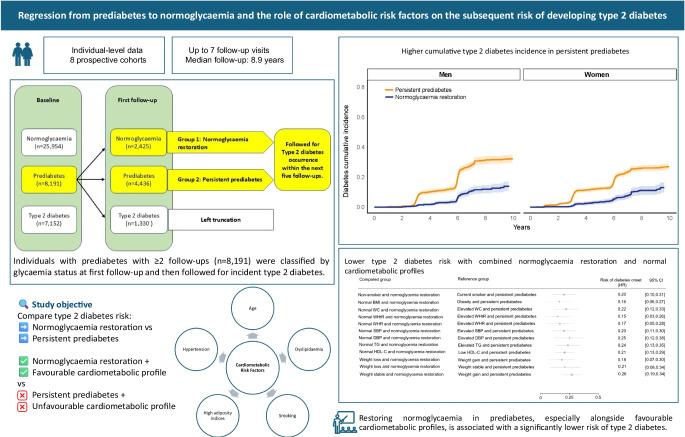

**Supplementary Information:**

The online version contains peer-reviewed but unedited supplementary material available at 10.1007/s00125-025-06555-8.


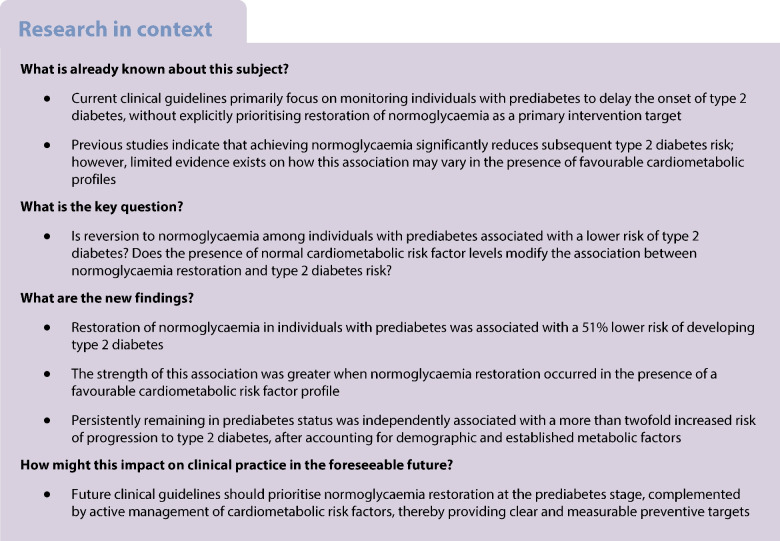


## Introduction

Type 2 diabetes is characterised by hyperglycaemia resulting from insulin deficiency and/or insulin resistance [1], and is one of the most prevalent chronic diseases globally, causing life-threatening complications [2]. An estimated 537 million adults worldwide are living with type 2 diabetes [3], which represents a growing health and economic burden [4, 5]. While genetic predisposition plays a role, exposure to cardiometabolic risk factors (CMRFs) and lifestyle behaviours significantly contribute to its development [6].

Type 2 diabetes has a subclinical stage known as prediabetes (intermediate hyperglycaemia), defined as fasting plasma glucose (FPG) 5.6–6.9 mmol/l in the absence of other criteria for type 2 diabetes, characterised by having raised levels of blood glucose that fall below the diagnostic threshold for type 2 diabetes, and its prevalence is projected to affect over 470 million people by 2030 [7]. Although individuals with prediabetes are at an increased risk of type 2 diabetes, with progression rates ranging from 2% to 50% annually [8], they may also regress to normoglycaemia [9]. Lifestyle interventions, including dietary modification, physical activity and weight loss, have shown efficacy in promoting glycaemic remission and delaying type 2 diabetes onset in the prediabetes population [10, 11]. The potential role of this regression in type 2 diabetes prevention has recently been highlighted [12–14]. Given that modifying CMRFs supports both regression from prediabetes and prevention of its progression, and that each pathway may independently reduce type 2 diabetes risk, it remains unclear whether the simultaneous achievement of normoglycaemia and optimal cardiometabolic profiles confers greater risk reduction. A previous study showed that among prediabetes individuals, achieving glycaemic remission alongside weight loss was associated with a significantly lower type 2 diabetes risk compared with weight loss alone [15]. These findings suggest that integrating glycaemic targets with weight loss goals may offer superior protection against type 2 diabetes [13]. However, the potential added value of addressing broader modifiable risk factors, such as smoking, lipid levels and blood pressure, remains underexplored.

To address this knowledge gap, and recognising that prediabetes represents a critical window for the primary prevention of type 2 diabetes and its associated complications [11], we hypothesised that (1) individuals with prediabetes who achieved normoglycaemia would have a lower risk of developing type 2 diabetes compared with those who remained in a prediabetes state; and (2) the combined normoglycaemia restoration and the presence of normal CMRF levels would be associated with an even lower risk of subsequent type 2 diabetes.

## Methods

### Study design and population

The Obesity, Diabetes and Cardiovascular Disease Collaboration (ODCDC) consortium is an international data pooling collaboration established to address outstanding issues of epidemiological and clinical importance regarding simple measures of obesity and the risk of incident type 2 diabetes in diverse populations [16]. We initially evaluated all 19 cohorts within the ODCDC and included seven studies with available data on baseline glucose status and at least two follow-ups. We also included the Atherosclerosis Risk in Communities (ARIC) study from the National Heart, Lung and Blood Institute (NHLBI) Biologic Specimen and Data Repository Information Coordinating Centre (BioLINCC) [17, 18]. The included studies were from the USA (*n*=3), Australia (*n*=2), Japan (*n*=2) and Iran (*n*=1).

**Fig. 1:**
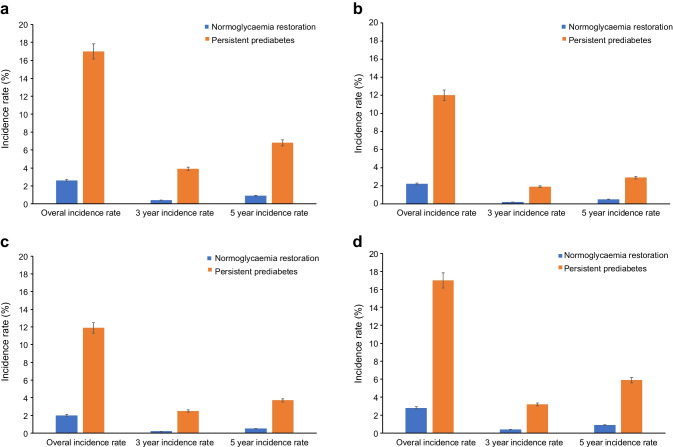
Gender- and age-specific unadjusted incidence rates (incident cases per 1000 person-years) of type 2 diabetes in the restoration of the normoglycaemia group and the persistent prediabetes group in (**a**) male participants, (**b**) female participants, (**c**) participants aged under 55 years, and (**d**) participants aged 55 years and older categories. The overall incidence rate, 3 year incidence rate and 5 year incidence rate are shown. Participants with follow-up periods closest to 3 years were categorised into the ‘3 year incidence’ group, while those with data extending beyond 3 years but not exceeding 5 years were included in the ‘5 year incidence’ group

Individuals with prediabetes at baseline were included in this study. Based on the glucose status at the first follow-up, we classified the study individuals into three groups: 1) the restoration of normoglycaemia group (individuals with prediabetes at baseline who achieved normoglycaemia at the first follow-up); 2) the persistent prediabetes group (individuals with prediabetes at baseline and the first follow-up); and 3) individuals with prediabetes at baseline who progressed to type 2 diabetes at the first follow-up (i.e. the left-truncated group). Participants in the normoglycaemia restoration and persistent prediabetes groups were subsequently followed to assess type 2 diabetes onset (electronic supplementary material [ESM] Fig. 1).

The selected eight cohorts included 9742 individuals with prediabetes at baseline. Of these, 1551 participants were excluded due to having fewer than two follow-up assessments. Among the remaining 8191 individuals with prediabetes who had glycaemic status data available from at least two follow-up visits (median intervals of 2.9 years [IQR 2.3–3.6] and 3.1 years [IQR 2.6–3.6]), 1330 progressed to type 2 diabetes at the first follow-up and were accounted for as left-truncated observations, while 2425 regressed to normoglycaemia and 4436 remained in a prediabetes state (ESM Fig. 2).

Ethics approval for this project was obtained from the Deakin University Human Research Ethics Committee (project number 2020-376). All included cohorts received approval from their respective institutional review boards or ethics committees, and written or oral informed consent was obtained from all participants. All procedures were conducted in accordance with the ethical standards of the institutional and national research committees and with the principles of the Declaration of Helsinki, as revised in 2024 (https://www.wma.net/policies-post/wma-declaration-of-helsinki-ethical-principles-for-medical-research-involving-human-subjects/).

### Ascertainment of prediabetes and type 2 diabetes

We categorised participants at each timepoint to one of the three following states based on the FPG, 2 h plasma glucose and/or HbA_1c_ cut-points recommended by the latest version of the ADA guidelines [19]. Type 2 diabetes was defined as an FPG of ≥7.0 mmol/l (126 mg/dl), 2 h plasma glucose of ≥11.1 mmol/l (200 mg/dl), HbA_1c_ ≥48 mmol/mol (6.5%), self-report type 2 diabetes, glucose-lowering medication use, or type 2 diabetes diagnosed in previous timepoints. Prediabetes was defined as an FPG 5.6–6.9 mmol/l (100–125 mg/dl) and not meeting other type 2 diabetes criteria, and normoglycaemia was defined as an FPG <5.6 mmol/l (100 mg/dl) and not meeting other type 2 diabetes criteria.

### Harmonisation of variable definitions and measurements across cohorts

To ensure the internal validity of our findings, rigorous harmonisation procedures were applied to variable definitions and measurements across all included cohorts. We considered age as both a continuous variable and a categorical variable (aged under 55 years and aged 55 years and older). A self-reported family history of type 2 diabetes in first-degree relatives was denoted by Yes and No. Smoking status was defined as current smoker, ex-smoker or non-smoker. BMI was calculated by dividing weight (in kilograms) by the square of height (in metres). We used the WHO cut-points to categorise it as normal weight (BMI <25 kg/m^2^), overweight (25 ≤ BMI <30 kg/m^2^) or obesity (BMI ≥30 kg/m^2^) [20]. For the two cohorts from Japan, we applied the WHO Asian BMI cut-points: normal weight (18.5–22.9 kg/m^2^), overweight (23–27.5 kg/m^2^) or obesity (BMI ≥27.5 kg/m^2^) [21]. Waist circumference (WC) was categorised into two groups (Normal, Elevated) according to ethnic-specific cut-off points for each cohort (ESM Table 1). WHR was calculated by dividing WC (cm) by hip circumference (cm). Likewise, the waist/height ratio (WHtR) was calculated by dividing WC (cm) by height (cm). The WHR and WHtR were categorised by WHO cut-off points, which were elevated for males with a WHR ≥0.9, females with a WHR ≥0.85, and a WHtR >0.5 for both, and otherwise normal [20]. Systolic blood pressure (SBP) and diastolic blood pressure (DBP) were classified as normal (SBP<130 mmHg, DBP<80 mmHg) and elevated (SBP≥130 mmHg, DBP≥80 mmHg, or antihypertensive drug use) [22]. Triacylglycerol level was categorised as normal (<1.7 mmol/l [<150 mg/dl]) and elevated (≥1.7 mmol/l [≥150 mg/dl]). HDL-cholesterol was categorised as low for male participants with HDL-cholesterol ≤1.0 mmol/l (≤40 mg/dl) and for female participants with HDL-cholesterol ≤1.3 mmol/l (≤50 mg/dl), and otherwise normal [23]. Region was classified according to WHO regional groupings: Americas, Western Pacific, and Eastern Mediterranean [24] (ESM Table 2). Weight change was defined as the percentage difference between the first and second follow-ups and categorised as ≥5% weight loss, stable weight (<5% loss and <2% gain) and ≥2% weight gain [13, 25].

We conducted a complete case analysis, as all covariates had less than 4% missing data (ESM Fig. 3).

### Statistical analysis

The characteristics of the study population at the first follow-up were summarised using mean and SD for continuous variables and frequency (%) for categorical variables. Survival analysis was performed to compare the age-adjusted cumulative incidence of type 2 diabetes between the restoration of the normoglycaemia group and the persistent prediabetes group.

We calculated the gender- and age-specific unadjusted incidence rates (incident cases per 1000 person-years) of type 2 diabetes for the restoration of the normoglycaemia group and the persistent prediabetes group. The incidence rates were determined for the 3 year and 5 year periods, as well as for the entire study duration. Participants with follow-up periods closest to 3 years were categorised into the ‘3 year incidence’ group, while those with data extending beyond 3 years but not exceeding 5 years were included in the ‘5 year incidence’ group.

To investigate the role of baseline FPG level in individuals with prediabetes at baseline on achieving normoglycaemia or remaining in the prediabetes state at the first follow-up, we explored the rates of normoglycaemia restoration and persistent prediabetes based on quartiles of baseline FPG levels.

To determine the role of CMRFs on type 2 diabetes risk, hierarchical mixed-effect proportional hazard Weibull (HMPHW) regression models were used. The models account for left truncation (i.e. the occurrence of type 2 diabetes prior to reversion to normoglycaemia at the first follow-up) using maximum likelihood estimation [26, 27]. The within-cohort clustering effect was accounted for by employing a hierarchical mixed-effect model that included a random intercept and a random-slope for each cohort. The estimated variance of the random effect term between eight cohorts in our HMPHW model with random intercept/slope was 1.9, indicating significant variability between included cohorts.

We developed a directed acyclic graph based on the literature review. The directed acyclic graph identifies interrelationships among covariates and type 2 diabetes risk (ESM Fig. 4). Therefore, the HMPHW model included age, gender, age and gender two-way interaction, family history of type 2 diabetes, smoking status, SBP and DBP, adiposity indices (BMI, WC, WHtR, WHR, body weight change), serum HDL-cholesterol and triacylglycerol levels. To mitigate the effect of multicollinearity, the role of each adiposity index was explored in a separate model. As indicated by previous studies that showed an inverse association between cigarette smoking and BMI [28], we examined the two-way interaction between smoking status with BMI.

All covariates were treated as time-fixed at the first follow-up, except body weight change, which incorporated data from both the first and second follow-ups. While gender and the interaction between age and gender were not statistically significant, we retained age, gender, and two-way interaction in the multivariable model due to their importance as potential effect modifiers of type 2 diabetes. Two-way interactions of all significant variables with the restoration of normoglycaemia were investigated in additional models.

Statistical analyses were carried out in R (R version 4.1.3) and Stata 18.

### Subgroup analyses

We conducted the following three subgroup analyses. First, we calculated the simultaneous association of the normoglycaemia restoration and having normal CMRF levels using HMPHW models. Second, we determined whether the role of CMRFs on the risk of type 2 diabetes was influenced by baseline FPG levels by performing a post hoc subgroup analysis by dividing participants into the lower quartiles (Q1 & Q2) and higher quartiles (Q3 & Q4). Third, we explored the potential sources of heterogeneity by conducting region-specific, random-effects meta-analysis by WHO region.

### Sensitivity analyses

We conducted the following sensitivity analyses. First, to mitigate the bias arising from defining type 2 diabetes based on a single abnormal measurement, which is common in observational studies, we defined type 2 diabetes based on two abnormal FPG (FPG ≥7.0 mmol/l (126 mg/dl)) test results obtained at two different timepoints [29], self-reported type 2 diabetes, glucose-lowering medication use or type 2 diabetes diagnosis at previous timepoints. FPG was considered because all included cohorts measured FPG. Second, to account for the competing risk of death, we performed multivariable analyses using competing risk regression, with death during follow-up as a competing event. We derived subhazard ratios (SHRs) and their 95% CIs from the multivariable-adjusted Fine–Grey model. Third, we modelled baseline FPG as a continuous variable (per 0.1 mmol/l) to estimate its association with type 2 diabetes risk.

## Results

Of the 8191 participants with prediabetes at baseline, 2425 (29.6%) individuals restored normoglycaemia, 4436 (54.2%) individuals remained in the prediabetes state, and 1330 (16.2%) individuals developed type 2 diabetes at first follow-up over a median follow-up of 8.9 years (IQR 3.1–14.8) (ESM Fig. 1). Gender-specific characteristics of the included sample are presented in Table 1, and cohort-specific characteristics are provided in ESM Tables 3 and 4. In brief, 59.4% of participants were male, the mean (SD) age of male participants was 53.6 (9.0) years and for female participants, 55.0 (8.8) years. At the first follow-up, the persistent prediabetes group showed higher mean BMI, WC and SBP compared with the group that regressed to normoglycaemia.

**Table 1:** Characteristics of the study population stratified by gender and glycaemic status

Characteristics	Male participants		Female participants	
	Normoglycaemia restoration	Persistent prediabetes	Normoglycaemia restoration	Persistent prediabetes
Number of participants	1480	2701	945	1735
Age in years	54.6 (10.2)	56.9 (7.8)	57.4 (9.6)	58.1 (7.8)
Smoking status, n (%)^a^				
Non-smoker	604 (40.8%)	919 (34.1%)	601 (63.9%)	1045 (60.3%)
Ex-smoker	514 (34.7%)	1175 (43.6%)	219 (23.3%)	402 (23.2%)
Current smoker	354 (23.9%)	601 (22.3%)	120 (12.8%)	285 (16.5%)
Adiposity indices				
BMI (kg/m^2^)	26.4 (4.2)	27.5 (4.1)	28.0 (5.7)	29.7 (6.9)
WC (cm)	94.6 (12.2)	98.5 (11.6)	92.9 (13.6)	99.0 (15.2)
WHtR	0.5 (0.1)	0.6 (0.1)	0.6 (0.1)	0.6 (0.1)
WHR	1.0 (0.1)	1.0 (0.1)	0.9 (0.1)	0.9 (0.1)
Weight change groups, n (%)^b^				
Weight loss	156 (10.8%)	282 (10.5%)	110 (12.0%)	234 (13.7%)
Weight stable	776 (53.6%)	1453 (54.2%)	389 (42.3%)	765 (44.7%)
Weight gain	517 (35.6%)	946 (35.3%)	420 (45.7%)	714 (41.7%)
SBP (mmHg)	126.4 (16.3)	128.8 (16.6)	125.5 (20.4)	128.9 (21.4)
DBP (mmHg)	77.8 (10.9)	78.4 (11.1)	71.9 (11.4)	72.8 (11.8)
Laboratory values				
Triacylglycerols (mmol/l)	1.6 (1.1)	1.7 (1.2)	1.4 (0.8)	1.6 (1.1)
HDL-cholesterol (mmol/l)	1.3 (0.4)	1.3 (0.4)	1.5 (0.4)	1.4 (0.4)
HbA_1c_ (mmol/mol), median (IQR)	34.9 (33.3–38.9)	38.9 (36.4–41.0)	35.7 (34.4–38.9)	38.9 (36.4–41.0)
HbA_1c_ (%), median (IQR)	5.3 (5.2–5.7)	5.7 (5.5–5.9)	5.4 (5.3–5.7)	5.7 (5.5–5.9)
FPG (mmol/l), median (IQR)	5.30 (5.10–5.49)	6.05 (5.80–6.38)	5.30 (5.10–5.44)	6.05 (5.82–6.38)

Data are presented as mean (SD) unless otherwise stated

^a^Participants with missing data were excluded

^b^Weight change was defined as the percentage difference between the first and second follow-ups and categorised as ≥5% weight loss, stable weight (<5% loss and <2% gain) and weight gain (≥2%)

The baseline characteristics of prediabetes individuals prior to classifications showed modest differences, with those who later restored normoglycaemia generally exhibiting a slightly more favourable cardiometabolic profile (ESM Table 5). The characteristics of the left-truncated group revealed a higher mean age, BMI, WHtR, WC, SBP and DBP, along with lower HDL-cholesterol, compared with the prediabetes population at first follow-up in both genders (ESM Table 6).

### Transition from prediabetes to other states of glycaemia

Compared with the group that transitioned back from prediabetes to normoglycaemia over the entire study period, as well as within the 3 year and 5 year follow-up durations, the age- and gender-specific incidence of type 2 diabetes were higher in the persistent prediabetes group. A comparison of gender-specific incidence rates between male and female participants demonstrated a higher incidence rate in male than female participants in both normoglycaemia restoration and persistent prediabetes groups. Similarly, a comparison of age-specific incidence rates between individuals under 55 years and those 55 years and older revealed a higher incidence rate in the older population across both groups (Fig. 1).

Our study showed that participants in the lowest quartile (Q1) of FPG at baseline exhibited nearly equal probabilities of restoring normoglycaemia (51.2%) and remaining in the prediabetes state (48.8%). However, as baseline glucose levels increased, the likelihood of remaining in the prediabetes state also increased. In the highest quartile (Q4), 80.7% of participants persisted in the prediabetes state, whereas only 19.3% restored normoglycaemia (ESM Fig. 5).

### Cumulative incidence of type 2 diabetes by glycaemic status

**Fig. 2:**
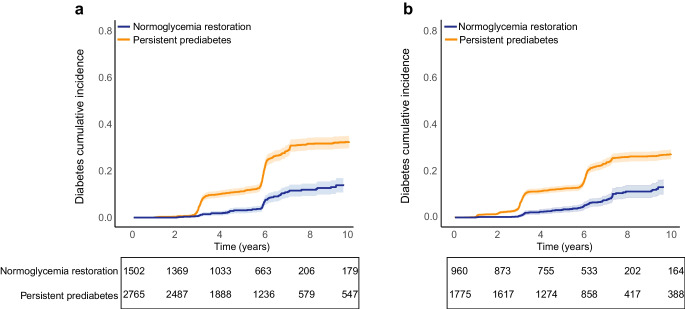
Age-adjusted cumulative incidence of type 2 diabetes among individuals with prediabetes who either reverted to normoglycaemia or remained in the prediabetes state, accounting for the competing risk of death in (**a**) male and (**b**) female participants. The shaded areas show 95% CIs. The numbers of individuals at each stage are shown below each graph

Compared with the group that transitioned back to normoglycaemia, the age-adjusted cumulative incidence of type 2 diabetes was higher in the persistent prediabetes group. The cumulative incidence curve from sensitivity analyses also demonstrated a similar trend (Fig. 2, ESM Fig. 6). The results from the HMPHW model showed that achieving normoglycaemia was associated with a 51% lower type 2 diabetes risk compared with persistent prediabetes (HR 0.49, 95% CI 0.42, 0.57; ESM Table 7). The HR decreased to 0.26 (0.16, 0.42) when type 2 diabetes was defined based on two abnormal FPGs (ESM Table 8), and SHR increased to 0.61 (0.42, 0.89) when considering the competing risk of death (ESM Table 9). In addition, when comparing the SHRs for the lower two quartiles (Q1 & Q2) and the upper two quartiles (Q3 & Q4) of FPG at baseline for participants who achieved normoglycaemia and individuals with persistent prediabetes, the adjusted type 2 diabetes risk was not materially different across groups and consistent with our main analysis results: SHR 0.47 (0.37, 0.58) for Q1 & Q2 and 0.48 (0.38, 0.59) for Q3 & Q4 (ESM Table 10). When modelled continuously, each 0.1 mmol/l increase in baseline FPG was associated with a 16% higher type 2 diabetes risk (ESM Table 7). Stratified analyses by WHO region demonstrated a similarly lower type 2 diabetes risk associated with normoglycaemia restoration: HR 0.45 (Americas), 0.29 (Western Pacific), and 0.44 (Eastern Mediterranean), and a pooled HR of 0.39 (0.29, 0.49), with considerable heterogeneity between regions (I^2^ = 81.62%; ESM Fig. 7).

### Effect modification by risk factors

**Fig. 3:**
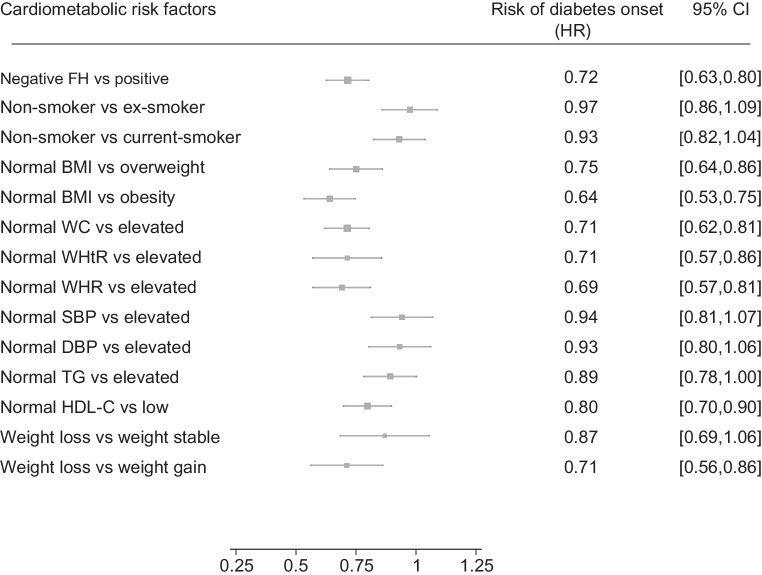
Model-adjusted HRs and 95% CIs for cardiometabolic risk factors associated with a lower risk of type 2 diabetes. Comparisons were made between favourable and unfavourable levels of each risk factor, adjusted for age, gender, self-reported family history of type 2 diabetes in first-degree relatives, smoking status, SBP and DBP, adiposity indices (BMI, WC, WHtR, WHR), serum HDL-cholesterol and triacylglycerol levels. The values of these predictors are derived from measurements obtained during the first follow-up. To mitigate the effect of multicollinearity, the role of each adiposity index was explored in a separate model. Weight change was defined as the percentage difference between the first and second follow-ups and categorised as ≥5% weight loss, stable weight (<5% loss and <2% gain) and weight gain (≥2%). FH, self-reported family history of type 2 diabetes in the first-degree relative; HDL-C, HDL-cholesterol; TG, triacylglycerol

Several factors were associated with a lower risk of type 2 diabetes. A negative family history of type 2 diabetes was associated with a 28% (95% CI 20%, 37%) lower type 2 diabetes risk compared with those with a positive history. Those with a normal BMI had a 25% (14%, 36%) and 36% (25%, 47%) lower type 2 diabetes risk compared with individuals classified as overweight or obese, respectively. Similar protective associations were observed with normal WHtR and WHR. Individuals with weight loss had a 29% (14%, 44%) lower type 2 diabetes risk compared with those who gained weight. Additionally, participants with normal HDL-cholesterol levels had a 20% (10%, 30%) lower risk compared with those with low HDL-cholesterol (Fig. 3). The results of subgroup analysis when comparing FPG quartile Q1 & Q2 vs Q3 & Q4 indicated a stronger protective association between normal adiposity indices and type 2 diabetes risk among individuals with lower FPG levels at baseline (ESM Table 10).

**Fig. 4:**
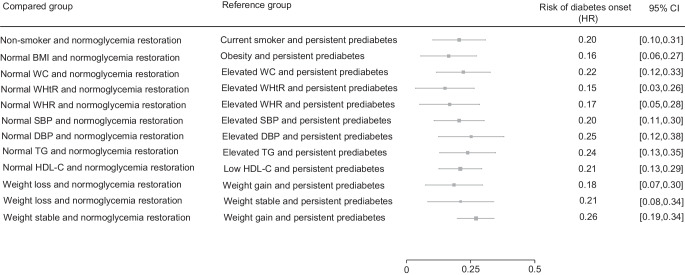
Model-adjusted HRs and 95% CIs for the combined association of normoglycaemia restoration and normal cardiometabolic risk factor level, compared with persistent prediabetes and unfavourable cardiometabolic profile, on the risk of developing type 2 diabetes. The model is adjusted for age, gender, self-reported family history of type 2 diabetes in the first-degree relatives, smoking status, SBP and DBP, adiposity indices (BMI, WC, WHtR, WHR), serum HDL-cholesterol and triacylglycerol levels. The values of these predictors are derived from measurements obtained during the first follow-up. To mitigate the effect of multicollinearity, the role of each adiposity index was explored in a separate model. Weight change was defined as the percentage difference between the first and second follow-ups and categorised as ≥5% weight loss, stable weight (<5% loss and <2% gain) and weight gain (≥2%). FH, self-reported family history of type 2 diabetes in the first-degree relative; HDL-C, HDL-cholesterol; TG, triacylglycerol

Subgroup analyses showed that achieving normoglycaemia, accompanied by normal CMRF levels, was associated with a lower type 2 diabetes risk. Non-smoker participants who achieved normoglycaemia had an 80% (69%, 90%) lower type 2 diabetes risk compared with current smokers with persistent prediabetes. An 84% (73%, 94%) lower type 2 diabetes risk was observed among participants with normal weight and normoglycaemia restoration, compared with those with obesity and persistent prediabetes. Similarly, having normal WC, WHtR and WHR were associated with 78% (67%, 88%), 85% (74%, 97%) and 83% (72%, 95%) lower type 2 diabetes risk, respectively, compared with those with elevated measurements. An 82% (70%, 93%) lower type 2 diabetes risk was observed among participants with both weight loss and normoglycaemia restoration, compared with those with weight gain and persistent prediabetes; those with stable weight showed 74% (66%, 81%) lower risk. Participants with normal SBP and DBP who returned to normoglycaemia had 80% (70%, 89%) and 75% (62%, 88%) lower type 2 diabetes risk, respectively. Additionally, the combination of normoglycaemia restoration and normal levels of triacylglycerols and HDL-cholesterol was associated with 76% (65%, 87%) and 79% (71%, 87%) lower type 2 diabetes risk, respectively (Fig. 4 and ESM Fig. 8).

The models’ goodness of fit was evaluated by plotting the deviance residuals against time. Deviance residuals were inspected to identify any misspecification or outliers with respect to the Weibull model. Additionally, violations of the proportional hazards’ assumption were examined using a Cox-Snell residual plot.

## Discussion

This pooled analysis of 6861 adults with prediabetes, the largest to date, demonstrated that achieving normoglycaemia was associated with over 50% lower type 2 diabetes risk, with an additional lower risk observed among those who also had a favourable cardiometabolic profile. The significance of normoglycaemia restoration is better understood when considering another aspect of our results. Remaining in a prediabetes state, compared with achieving normoglycaemia, was associated with at least a twofold higher type 2 diabetes risk, independent of age, gender and established CMRFs including smoking, overweight or obesity, dyslipidaemia and hypertension. Hence, prediabetes represents a critical state for the prevention of type 2 diabetes, during which normoglycaemia restoration and optimal management of comorbid conditions can be most effectively implemented.

Despite the high prevalence of prediabetes and literature which suggests that around 70% of individuals living with prediabetes eventually progress to type 2 diabetes if no intervention is taken [30, 31], the long-term potential benefits of achieving normoglycaemia in the prediabetes population have not been sufficiently elucidated [32]. Limitations such as sample size, an insufficient follow-up duration, lack of multiple follow-ups, and technical difficulties in dealing with left truncation due to conversion to type 2 diabetes were potential barriers in previous research. In our study, we addressed these issues by including eight community-dwelling prospective cohorts to increase the sample size, incorporating up to seven follow-up assessments per cohort, and employing HMPHW to handle left truncation.

In this study, although the direction of association between normoglycaemia restoration and lower type 2 diabetes risk was consistent across regions, the magnitude varied, reflecting differences in population characteristics, baseline diabetes risk, healthcare access and study design [33]. Regional heterogeneity in ethnicity, socioeconomic status, diagnostic thresholds, screening intensity and follow-up duration probably influenced both the probability of glycaemic regression and the underlying type 2 diabetes risk [5, 34]. Additionally, population-level differences in pathophysiological mechanisms, such as variation in insulin resistance, beta cell function, fat distribution and the prevalence of specific diabetes subtypes, may influence how individuals respond to metabolic improvements [35]. These disparities, shaped by complex host–environment–lifestyle interactions and varying access to preventive care, contribute to the observed differences in effect estimates across regions [33].

Individuals living with prediabetes are typically managed through lifestyle interventions, which are considered first-line treatment [10]. These interventions include dietary modifications alongside regular physical activity aimed at improving insulin sensitivity and glycaemic management [10, 14]. Pharmacological treatments, such as metformin, may also be prescribed for individuals at higher risk of progression to type 2 diabetes, particularly those unable to achieve sufficient lifestyle changes [10]. Our analysis further supports this approach by demonstrating that restoration of normoglycaemia in individuals with prediabetes is associated with a significantly lower type 2 diabetes risk, particularly when accompanied by favourable cardiometabolic profiles, including normal blood pressure, serum HDL-cholesterol and triacylglycerol levels, healthy weight and non-smoking status. Therefore, our findings suggest that the combination of normoglycaemia restoration and normal CMRF levels is associated with a substantially lower type 2 diabetes risk in prediabetes individuals. This metabolic profile may lower the long-term burden of type 2 diabetes by preserving beta cell function and improving insulin sensitivity [12, 36, 37].

Our findings highlight the complex association between smoking history and type 2 diabetes risk. Although this association was non-significant in the primary analysis, subgroup analyses suggested that both non-smokers and ex-smokers who achieved normoglycaemia had a significantly lower type 2 diabetes risk compared with current smokers with persistent prediabetes. These findings highlight the potential benefits of smoking cessation and glycaemic management in reducing type 2 diabetes risk. Interestingly, the association was non-significant among ex-smokers who did not achieve normoglycaemia, which may be influenced by factors such as weight gain following smoking cessation. These insights emphasise the need for continued public health efforts to support smoking cessation and effective glycaemic management as part of type 2 diabetes prevention strategies. We also recommend post-cessation weight management to enhance the beneficial effects of smoking cessation on type 2 diabetes prevention.

In our study, normal adiposity indices were associated with a significantly lower type 2 diabetes risk, aligning with findings from previous research on Japanese populations that highlighted the role of lifestyle modifications in preventing type 2 diabetes among middle-aged, overweight individuals with prediabetes [38]. We also observed that the association between normal adiposity indices and lower type 2 diabetes risk was stronger among individuals with lower baseline FPG levels compared with those with higher baseline levels. Subgroup analyses indicated that achieving normoglycaemia while being categorised as overweight was associated with a 61% lower type 2 diabetes risk, compared with individuals with persistent prediabetes and obesity. However, the type 2 diabetes risk associated with being overweight remains 24% higher than that observed in individuals with a normal weight. Consistently, evidence from previous studies indicates that achieving prediabetes remission through lifestyle-induced weight loss of over 7% significantly improves insulin sensitivity and beta cell function, resulting in a 73% reduction in the type 2 diabetes risk [11, 13, 15]. The results of our study suggest that while weight loss is associated with a lower type 2 diabetes risk in prediabetes individuals, achieving a normal weight may not always be necessary to observe a beneficial effect. These results suggest the importance of a dual approach that targets both weight management and normoglycaemia restoration as a strategy to effectively reduce the type 2 diabetes risk in prediabetes individuals.

The significantly lower type 2 diabetes risk among prediabetes individuals who achieved normoglycaemia is particularly important when considering that conditions such as hypertension, obesity and dyslipidaemia – common risk factors for CVD – often accompany type 2 diabetes. Thus, reducing type 2 diabetes risk may also decrease CVD risk, not only through the direct impact of lowering type 2 diabetes but also via concurrent improvements in other CMRFs [39]. Restoring normoglycaemia may therefore reduce the incidence of CHD, ischaemic stroke and vascular dementia [40–42]. Given that CVD is the leading cause of morbidity and mortality in individuals with type 2 diabetes [1, 39, 43], a lower type 2 diabetes risk may translate into a reduced burden of vascular complications.

A major strength of our study is the use of data from eight prospective cohorts, yielding a large and diverse sample that enabled robust multivariable modelling and improved generalisability across populations. Harmonised definitions and measurements enhanced internal validity by minimising between-cohort differences. Additionally, while single glucose measurements are commonly used in epidemiological research, our sensitivity analysis applied a stricter definition of type 2 diabetes based on two abnormal FPG values, aligning more closely with clinical diagnostic standards.

One of the limitations of the current study is the susceptibility to selection bias due to loss-to-follow-up over the waves of the included studies. Moreover, the lack of data on behavioural and lifestyle factors may have introduced residual confounding bias. Baseline differences in cardiometabolic profiles between individuals who restored normoglycaemia and those with persistent prediabetes suggest that the former group may have had an inherently lower risk profile, potentially amplifying the apparent benefit associated with normoglycaemia restoration. The absence of cohorts from Europe, South-East Asia and Africa, along with observed regional heterogeneity, may limit the generalisability of our findings across populations.

### Conclusion

Our results highlight the importance of normoglycaemia restoration in the prediabetes stage, as it is associated with a substantially lower risk of developing type 2 diabetes. To our knowledge, this is the first study to quantify the association between restoring normoglycaemia and concurrently having normal CMRF levels with a substantially lower type 2 diabetes risk in individuals with prediabetes. Therefore, we recommend considering the concept of restoring normoglycaemia in individuals with prediabetes in future guidelines, as it has the potential to reduce the risk of developing type 2 diabetes.

## Supplementary Information

Below is the link to the electronic supplementary material.

ESM (PDF 1.20 MB)

## Data Availability

De-identified individual participant data that underlie the findings reported in this manuscript will not be publicly available due to data governance agreements with the original cohort custodians. However, access to the data may be granted upon reasonable request for methodologically sound proposals and subject to approval by the ODCDC Steering Committee and the respective cohort governance committees. Researchers wishing to request data access should contact the corresponding author (n.davoodian@deakin.edu.au) with a detailed proposal outlining the research objectives, methods and planned use of the data.

## Abbreviations

CMRFs: Cardiometabolic risk factors
DBP: Diastolic blood pressure
FPG: Fasting plasma glucose
HMPHW: Hierarchical mixed-effect proportional hazards Weibull
SBP: Systolic blood pressure
SHR: Subhazard ratios
WC: Waist circumference
WHtR: Waist/height ratio
