# Supplementary material for: Regression from prediabetes to normoglycaemia and the role of cardiometabolic risk factors on the subsequent risk of developing type 2 diabetes
Source: Diabetologia. 2025 Oct 18;69(1):69–80. doi: 10.1007/s00125-025-06555-8 (PMC12686057; doi:10.1007/s00125-025-06555-8)
Supplement: Supplementary file 1 — ESM (PDF 1.20 MB) [file 125_2025_6555_MOESM1_ESM.pdf]

Supplementary appendix

Regression from prediabetes to normoglycaemia and the role of cardiometabolic risk factors on subsequent risk of developing type 2 diabetes

Contents list of supplementary material: CLICK on any item to jump to it.

Supplementary Tables

ESM Table 1. Ethnic-specific values for waist circumference........ 3
ESM Table 2. Distribution of Included Cohort Studies by WHO Region........ 4
ESM Table 3. Characteristics of included studies........ 5
ESM Table 4. Baseline characteristics of included cohort studies........ 6
ESM Table 5. Baseline characteristics of individuals with prediabetes prior to stratification by glycaemia status at first follow-up........ 7
ESM Table 6. Characteristics of the left truncated population stratified by gender........ 8
ESM Table 7. Predictors associated with the lower risk of type 2 diabetes mellitus........ 9
ESM Table 8. Predictors associated with the lower risk of type 2 diabetes obtained from sensitivity analysis 1........ 10
ESM Table 9. Predictors associated with the lower risk of type 2 diabetes obtained from sensitivity analysis 2........ 11
ESM Table 10. Predictors associated with the lower risk of type 2 diabetes obtained from sensitivity analysis 2, stratified by quartile of fasting plasma glucose at baseline... 12

Supplementary Figures

ESM Fig. 1. Schematic visualization of study group classification........ 13
ESM Fig. 2. Flow chart for pooling population cohorts and exclusions was made to form the study population samples........ 14
ESM Fig. 3. Missing data percentages across included cohorts, stratified by glycaemic status........ 15
ESM Fig. 4. Directed acyclic graph illustrating hypothesized causal pathways between risk factors and prediabetes progression to type 2 diabetes........ 16
ESM Fig. 5. Percentage of participants who restored normoglycaemia or remained in the prediabetes stage at first follow-up, by quartile of fasting plasma glucose at baseline. 17
ESM Fig. 6. Nelson-Aalen plot of age-adjusted total and gender-specific cumulative risk of type 2 diabetes in individuals with prediabetes, stratified by glucose status at the first follow-up into those who transitioned to normoglycaemia and those with persistent prediabetes. 18
ESM Fig. 7. Hazard Ratios for diabetes onset comparing individuals who restored normoglycaemia to those with persistent prediabetes across WHO Regions........ 19
ESM Fig. 8. Model-adjusted hazard ratios (HRs) extracted from subgroup analyses estimating the simultaneous effect of restoring normoglycaemia and having normal cardiometabolic risk factor levels on the risk of type 2 diabetes. 20

Acronym of included studies:

Aichi; Aichi workers cohort (1)

ARIC; Atherosclerosis Risk in Communities Study (2, 3)

AUSDIAB; Australian Obesity, Diabetes and Lifestyle Study (4)

JHS; Jackson Heart Study (5).

MESA; Multi-Ethnic Study of Atherosclerosis (6)

NWAHS; North West Adelaide Health Study (7)

TLGS; Tehran Lipids and Glucose Study (8)

TOYAMA; Toyama prefecture study (9)

ESM Table 1. Ethnic-specific values for waist circumference

Country/Ethnic Group	Waist circumference	Cohort Study
Europids [a] (10)		
Males	≥94 cm	AUSDIAB
Females	≥80 cm	NWAHS
North America (10)		
Males	≥102 cm	JHS
Females	≥88 cm	MESA
		ARIC
Japanese (11)		
Males	≥90 cm	TOYAMA
Females	≥80 cm	Aichi
Iranian (12)		
Males	≥90 cm	TLGS
Females	≥90 cm	

*In the USA, the ATP III values are likely to continue to be used for clinical purposes.

ESM Table 2. Distribution of Included Cohort Studies by WHO Region

WHO regions	Country	Cohort Study	Number of participants
**Region of the Americas**	USA	JHS<br>MESA<br>ARIC [a]	4986
**Western Pacific Region**	Australia and Japan	AUSDIAB<br>NWAHS<br>TOYAMA<br>Aichi	2727
**Eastern Mediterranean Region**	Iran	TLGS	395

[a] Atherosclerosis Risk in Communities Study Research Materials obtained from the NHLBI Biologic Specimen and Data Repository Information Coordinating Centre (BioLINCC).

ESM Table 3. Characteristics of included studies

Study abbreviation	Characteristics of studies					Follow-ups	
	Country	Participants			Baseline years	Median follow-up (Years)	N of visits
		Total	Male participants	Female participants			
**Aichi**	Japan	8,944	6,885	2,104	2002-2005	6.7	5
**ARIC** [a]	USA	14,832	6,723	7,970	1987-89	22	5
**AUSDIAB**	Australia	11,191	5,024	6,167	1999-2000	11.7	3
**JHS**	USA	5,306	1,935	3,371	2000-2004	15	3
**MESA**	USA	6,814	3,213	3,601	2000-2002	9.0	6
**NWAHS**	Australia	4,059	1,932	2,124	1999-2000, 2002-2003	7	3
**TLGS**	Iran	6,437	2,745	3,692	1999-2001	12.6	5
**TOYAMA**	Japan	6,926	4,651	2,275	2009	4.9	7

[a] Atherosclerosis Risk in Communities Study Research Materials obtained from the NHLBI Biologic Specimen and Data Repository Information Coordinating Centre (BioLINCC).

Abbreviations: Aichi, Aichi workers cohort; ARIC, Atherosclerosis Risk in Communities Study; AUSDIAB, Australian Obesity, Diabetes and Lifestyle Study; JHS, Jackson Heart Study; MESA, Multi-Ethnic Study of Atherosclerosis; NWAHS, North West Adelaide Health Study; TLGS, Tehran Lipids and Glucose Study; TOYAMA, Toyama prefecture study

ESM Table 4. Baseline characteristics of included cohort studies

Study abbreviation	Number	Age at baseline	Smoking status [n (%)]			BMI	WC (cm)	WHtR	WHR	SBP (mmHg)	DBP (mmHg)	TG (mmol/l)	HDL-C (mmol/l)
		Mean (SD)	Non - smoker	Ex-smoker	Current smoker	Mean (SD	Mean (SD)	Mean (SD)	Mean (SD)	Mean (SD)	Mean (SD)	Mean (SD)	Mean (SD)
**Aichi**	459	50.62 (5.9)	178 (43.1)	122 (29.54%)	113 (27.36%)	23.65 (2.92)	83.92 (7.92)	0.5 (0.05)		130.97 (14.86)	82.93 (11.13)	1.46 (0.93)	1.49 (0.38)
**ARIC** [a]	4,088	57.5 (5.6)	1613 (39.5%)	1588 (38.9%)	884 (21.6%)	28.8 (5.2)	100.8 (13.6)	0.6 (0.1)	0.9 (0.1)	122.6 (17.5)	74.6 (10.7)	1.6 (1.0)	1.3 (0.4)
**AUSDIAB**	1,157	56.4 (10.3)	593 (57.68%)	328 (31.9%)	107 (10.4%)	28.45 (4.63)	96.95 (12)	0.57 (0.07)	0.91 (0.08)	125.84 (17.47)	71 (9.71)	1.58 (0.99)	1.36 (0.36)
**JHS**	204	58.8 (10.0)	140 (63.9%)	56 (25.6%)	23 (10.5%)	33.5 (6.5)	106.4 (14.6)	0.6 (0.1)		130.0 (18.4)	74.3 (10.6)	1.3 (0.9)	1.4 (0.4)
**MESA**	694	65.3 (9.5)	49 (10.0%)	180 (36.6%)	263 (53.5%)	29.5 (5.3)	101.2 (13.7)	0.6 (0.1)	0.9 (0.1)	128.6 (20.6)	72.1 (10.3)	1.6 (1.1)	1.3 (0.3)
**NWAHS**	329	57.85 (12.74)	125 (44.48%)	101 (35.94%)	55 (19.57%)	29.43 (4.67)	99.46 (11.33)	0.59 (0.07)	0.92 (0.07)	132.46 (17.27)	83.75 (9.35)	1.91 (1.55)	1.34 (0.34)
**TLGS**	395	50.9 (12.8)	37 (13.3%)	19 (6.8%)	223 (79.9%)	29.0 (4.6)	98.0 (10.5)	0.6 (0.1)	0.9 (0.1)	124.2 (19.5)	78.8 (11.5)	2.1 (1.3)	1.0 (0.2)
**TOYAMA**	782	51.0 (6.9)	219 (31.5%)	157 (22.6%)	320 (46.0%)	24.2 (3.6)	86.3 (9.2)	0.5 (0.1)		125.7 (17.0)	79.1 (11.2)	1.4 (1.0)	1.5 (0.4)

[a] Atherosclerosis Risk in Communities Study Research Materials obtained from the NHLBI Biologic Specimen and Data Repository Information Coordinating Centre (BioLINCC).

Abbreviations: BMI, body mass index; DBP, diastolic blood pressure; SBP, systolic blood pressure; TG, Triacylglycerols; HDL-C, high-density lipoprotein cholesterol; WC, waist circumference; WHtR, waist height ratio; WHR, waist-hip ratio; Aichi, Aichi workers cohort; ARIC, Atherosclerosis Risk in Communities Study; AUSDIAB, Australian Obesity, Diabetes and Lifestyle Study; JHS, Jackson Heart Study; MESA, Multi-Ethnic Study of Atherosclerosis; NWAHS, North West Adelaide Health Study; TLGS, Tehran Lipids and Glucose Study; TOYAMA, Toyama prefecture study

ESM Table 5. Baseline characteristics of individuals with prediabetes prior to stratification by glycaemia status at first follow-up

	Normoglycaemia restoration	Persistent Prediabetes
Number of participants	2,425	4,436
Age (y) (mean ±SD)	53.1 (9.4)	54.2 (8.3)
Smoking status, n (%)		
Non-smoker	1,145 (47.2%)	2,050 (46.2%)
Ex-smoker	795 (32.8%)	1,548 (34.9%)
Current smoker	485 (20.0%)	838 (18.9%)
Adiposity indices		
BMI (kg/m2) (mean ±SD)	26.9 (4.7)	27.6 (4.8)
WC (cm) (mean ±SD)	93.7 (12.6)	95.1 (12.6)
WHtR (mean ±SD)	0.6 (0.1)	0.6 (0.1)
WHR (mean ±SD)	0.9 (0.1)	0.9 (0.1)
SBP (mmHg) (mean ±SD)	124.8 (17.6)	125.9 (17.7)
DBP (mmHg) (mean ±SD)	75.5 (11.2)	75.7 (11.1)
Laboratory values		
Triacylglycerols (mmol/l) (mean ±SD)	1.5 (1.0)	1.6 (1.0)
HDL cholesterol (mmol/l) (mean ±SD)	1.4 (0.4)	1.3 (0.4)
HbA1c (mmol/mol), median (IQR)	34.4 (31.6–36.6)	35.6 (33.1–38.8)
HbA1c (%), median (IQR)	5.3 (5.0–5.5)	5.4 (5.1–5.7)
FPG (mmol/L), median (IQR)	5.8 (5.7–5.9)	5.9 (5.7–6.1)

Abbreviations: BMI, body mass index; DBP, diastolic blood pressure; SBP, systolic blood pressure; WC, waist circumference; WHtR, waist height ratio; WHR, waist hip ratio; FPG, fasting plasma glucose.

ESM Table 6. Characteristics of the left truncated population stratified by gender.

	**Male participants (N=684)**	**Female participants (N=646)**
**Age at first follow-up (Mean, SD)**	58.9 (8.6)	58.6 (9.0)
**Non-smoker**	197 (34.1%)	327 (62.4%)
**Ex-smoker**	264 (45.7%)	119 (22.7%)
**Current smoker**	117 (20.2%)	78 (14.9%)
**BMI (Mean, SD)**	29.6 (4.9)	32.3 (7.2)
**WC (cm) (Mean, SD)**	104.3 (14.1)	104.8 (16.6)
**WHtR (Mean, SD)**	0.6 (0.1)	0.7 (0.1)
**WHR (Mean, SD)**	1.0 (0.1)	0.9 (0.1)
**SBP (mmHg) (Mean, SD)**	131.1 (17.7)	129.8 (20.9)
**DBP (mmHg) (Mean, SD)**	79.3 (11.0)	73.1 (11.6)
**TG (mmol/l) (Mean, SD)**	2.1 (1.5)	1.7 (0.9)
**HDL-C (mmol/l) (Mean, SD)**	1.2 (0.3)	1.3 (0.4)
Abbreviations: BMI, body mass index; DBP, diastolic blood pressure; SBP, systolic blood pressure; TG, Triacylglycerols; HDL-C, high-density lipoprotein cholesterol; WC, waist circumference; WHtR, waist height ratio; WHR, waist hip ratio		

ESM Table 7. Predictors associated with the lower risk of type 2 diabetes mellitus.

Prognostic factors	Hazard ratio	95% CI		P value
Restoration of normoglycaemia vs persistent prediabetes	0.49	0.42	0.57	<0.01
Baseline FPG (per 0.1 mmol/L increase)	1.16	1.12	1.20	<0.01
Gender	0.83	0.31	2.24	0.73
Negative FH vs positive	0.72	0.63	0.80	<0.01
Non-smoker vs ex-smoker	0.97	0.87	1.10	0.71
Non-smoker vs current smoker	0.93	0.83	1.00	0.04
Normal BMI vs overweight	0.75	0.64	0.86	<0.01
Normal BMI vs obesity	0.64	0.53	0.75	<0.01
Normal WC vs elevated	0.71	0.63	0.81	<0.01
Normal WHtR vs elevated	0.71	0.58	0.87	<0.01
Normal WHR vs elevated	0.69	0.57	0.81	<0.01
Normal SBP vs elevated	0.94	0.82	1.10	0.39
Normal DBP vs elevated	0.93	0.81	1.10	0.33
Normal TG vs elevated	0.89	0.79	1.01	0.07
Normal HDL-C vs low	0.80	0.70	0.9	<0.01

a) The hazard ratios (HRs) of predictors associated with the lower risk of type 2 diabetes were obtained from a hierarchical mixed-effect proportional hazards Weibull (HMPHW) model with random intercept and slope.
b) The model is adjusted for age, gender, age and gender two-way interaction, self-reported family history of type 2 diabetes in the first-degree relatives, smoking status, systolic-diastolic blood pressure, adiposity indices (BMI, WC, WHtR, WHR), serum HDL-C, and Triacylglycerols levels.
c) To mitigate the effect of multicollinearity, each adiposity index (BMI, WC, WHtR, WHR) was included in a separate model.
d) The estimated variance of the random effect term between eight cohorts in our HMPHW model with random intercept/slope was 1.9, indicating significant variability between included cohorts.
Abbreviations: BMI, body mass index; DBP, diastolic blood pressure; SBP, systolic blood pressure; TG, Triacylglycerols; HDL-C, high-density lipoprotein cholesterol; FH, family history of type 2 diabetes in the first-degree relative; FPG, fasting plasma glucose; HR, Hazard ratio.

ESM Table 8. Predictors associated with the lower risk of type 2 diabetes obtained from sensitivity analysis 1.

Prognostic factors	Hazard ratio	95% CI		P value
Restoration of normoglycaemia vs persistent prediabetes	0.26	0.16	0.42	<0.01
Gender	0.53	0.02	15.14	0.71
Negative FH vs positive	0.84	0.53	1.35	0.47
Non-smoker vs Ex-smoker	0.97	0.37	2.5	0.95
Non-smoker vs Current smoker	0.93	0.39	2.2	0.88
Normal BMI vs Overweight	0.73	0.45	1.20	0.22
Normal BMI vs Obesity	0.64	0.53	0.75	<0.01
Normal WC vs Elevated	0.66	0.42	1.02	0.06
Normal WHtR vs Elevated	0.48	0.25	0.92	0.03
Normal WHR vs Elevated	0.58	0.32	1.08	0.08
Normal SBP vs Elevated	0.69	0.44	1.07	0.09
Normal DBP vs Elevated	1.18	0.73	1.91	0.5
Normal TG vs Elevated	0.79	0.54	1.18	0.25
Normal HDL-C vs Low	0.81	0.52	1.25	0.34

a) Sensitivity analysis 1: Aligning the type 2 diabetes definition more closely with clinical standards.
b) The hazard ratios (HRs) of predictors associated with the reduction in the risk of type 2 diabetes were obtained from a hierarchical mixed-effect proportional hazards Weibull (HMPHW) model with random intercept and slope.
c) The model is adjusted for age, gender, age and gender two-way interaction, self-reported family history of type 2 diabetes in the first-degree relatives, smoking status, systolic-diastolic blood pressure, adiposity indices (BMI, WC, WHtR, WHR), serum HDL-C, and Triacylglycerols levels.
d) To mitigate the effect of multicollinearity, each adiposity index (BMI, WC, WHtR, WHR) was included in a separate model.
e) The estimated variance of the random effect term between eight cohorts in our HMPHW model with random intercept/slope was 2.7, indicating significant variability between included cohorts.
Abbreviations: BMI, body mass index; DBP, diastolic blood pressure; SBP, systolic blood pressure; TG, Triacylglycerols; HDL-C, high-density lipoprotein cholesterol; FH, family history of type 2 diabetes in the first-degree relative; HR, Hazard ratio.

ESM Table 9. Predictors associated with the lower risk of type 2 diabetes obtained from sensitivity analysis 2.

**Prognostic factors**	**Subhazard ratio**	**95% CI**		**P value**
Restoration of normoglycaemia vs persistent prediabetes	0.61	0.42	0.89	<0.01
Gender	0.90	0.67	1.2	0.49
Negative FH vs positive	0.82	0.59	1.13	1.66
Non-smoker vs Ex-smoker	0.67	0.33	1.35	0.26
Non-smoker vs Current smoker	0.90	0.34	2.30	0.83
Normal BMI vs Overweight	0.69	0.47	1.03	0.07
Normal BMI vs Obesity	0.58	0.38	0.87	<0.01
Normal WC vs Elevated	0.60	0.45	0.80	<0.01
Normal WHtR vs Elevated	0.66	0.40	1.06	0.09
Normal WHR vs Elevated	0.60	0.40	0.91	0.01

a) Sensitivity analysis 2: Considering the competing risk of death
b) Subhazard ratios were calculated from Fine-Gray models adjusted for age, gender, age and gender two-way interaction, self-reported family history of type 2 diabetes in the first-degree relatives, smoking status, and adiposity indices (BMI, WC, WHtR, WHR).
c) To mitigate the effect of multicollinearity, each adiposity index (BMI, WC, WHtR, WHR) was included in a separate model.
Abbreviations: BMI, body mass index; FH, family history of type 2 diabetes in the first-degree relative.

ESM Table 10. Predictors associated with the lower risk of type 2 diabetes obtained from sensitivity analysis 2, stratified by quartile of fasting plasma glucose at baseline.

**Prognostic factors**	**Q1 & Q2**		**Q3 &Q4**	
	**Subhazard ratio**	**95% CI**	**Subhazard ratio**	**95% CI**
Restoration of normoglycaemia vs persistent prediabetes	0.47	(0.37 - 0.58)	0.48	(0.38 - 0.59)
Gender	0.91	(0.75 - 1.11)	0.89	(0.77 - 1.02)
Negative FH vs positive	0.80	(0.65 - 0.98)	0.84	(0.72 - 0.99)
Non-smoker vs Ex-smoker	1.04	(0.85 - 1.27)	0.97	(0.82 - 1.14)
Non-smoker vs Current smoker	0.92	(0.72 - 1.18)	0.74	(0.61 - 0.89)
Normal BMI vs Overweight	0.61	(0.47 - 0.79)	0.90	(0.74 - 1.103)
Normal BMI vs Obesity	0.35	(0.26 - 0.45)	0.61	(0.50 - 0.75)
Normal WC vs Elevated	0.51	(0.42 - 0.62)	0.76	(0.66 - 0.88)
Normal WHtR vs Elevated	0.42	(0.30 - 0.59)	0.60	(0.46 - 0.79)
Normal WHR vs Elevated	0.42	(0.33 - 0.54)	0.49	(0.38 - 0.65)

a) Sensitivity analysis 2: Considering the competing risk of death
b) Q1 & Q2 represent the lower two quantiles of fasting plasma glucose at baseline, indicating lower glucose levels (5.6 - 5.9). Q3 & Q4 represent the upper two quantiles, indicating higher glucose levels (5.9 - 7.0).
c) Subhazard ratios were calculated from Fine-Gray models adjusted for age, gender, age and gender two-way interaction, self-reported family history of type 2 diabetes in the first-degree relatives, smoking status, and adiposity indices (BMI, WC, WHtR, WHR).
d) To mitigate the effect of multicollinearity, each adiposity index (BMI, WC, WHtR, WHR) was included in a separate model.

Abbreviations: BMI, body mass index; FH, family history of type 2 diabetes in the first-degree relative.

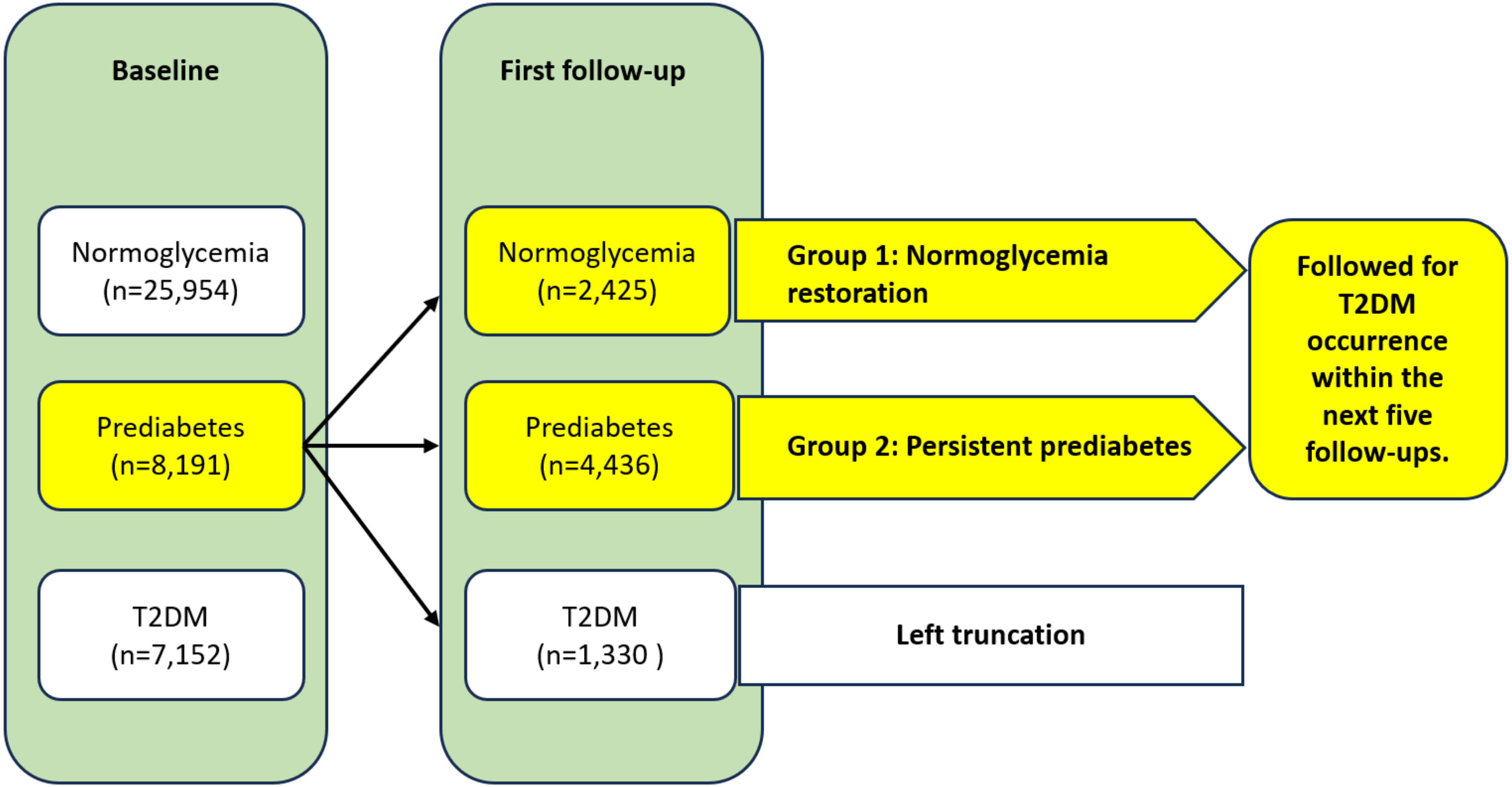

ESM Fig. 1. Schematic visualization of study group classification

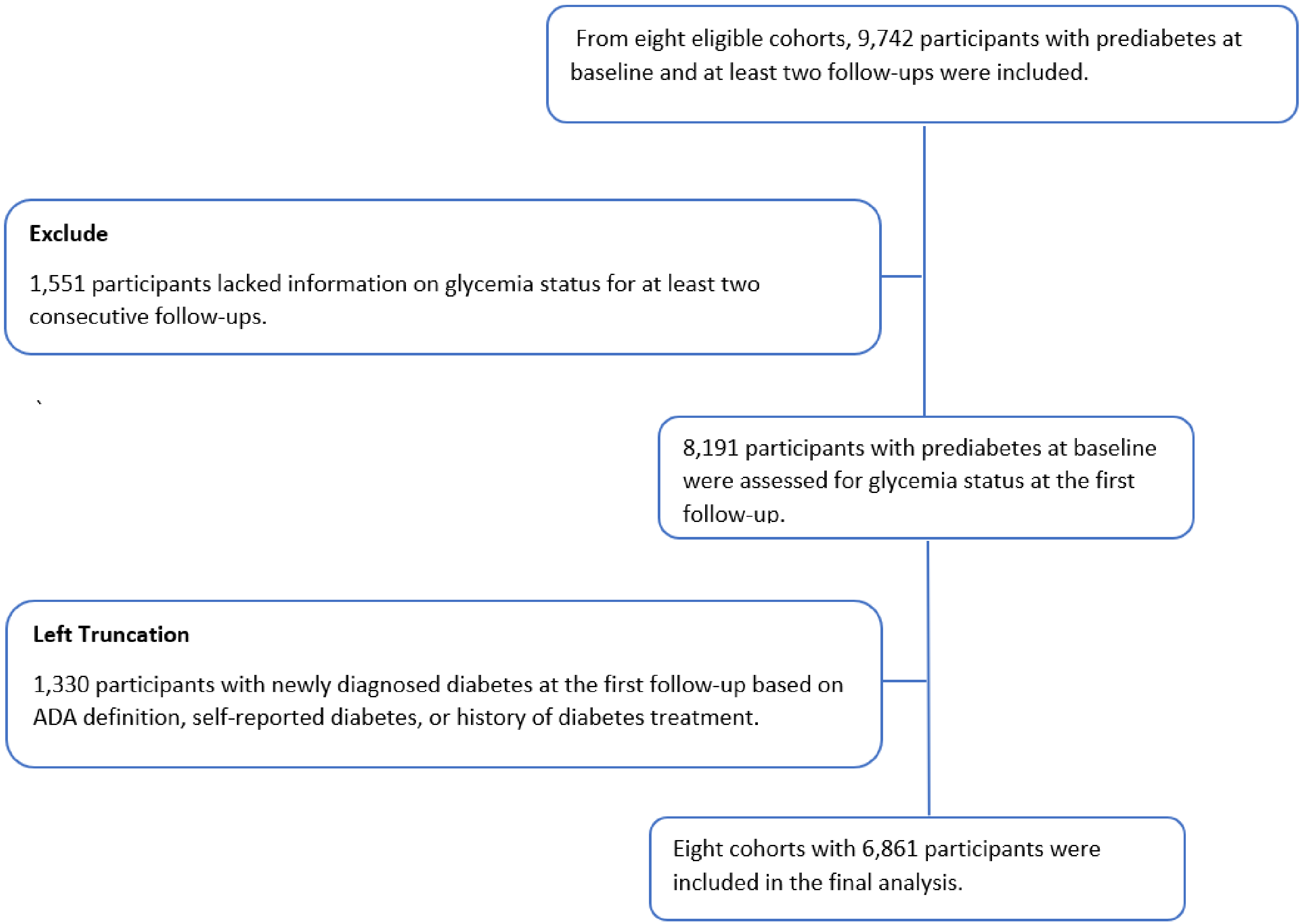

ESM Fig. 2. Flow chart for pooling population cohorts and exclusions was made to form the study population samples.

a) Seven prospective cohorts with accurate glucose status at baseline and at least two follow-ups and enough sample size from the Obesity, Diabetes, and Cardiovascular Disease Collaboration database were included.
b) Atherosclerosis Risk in Communities Study research materials obtained from the NHLBI Biologic Specimen and Data Repository Information Coordinating Centre (BioLINCC).
c) For the Robustness of our analysis four cohorts with less than 100 participants with prediabetes at baseline were excluded.

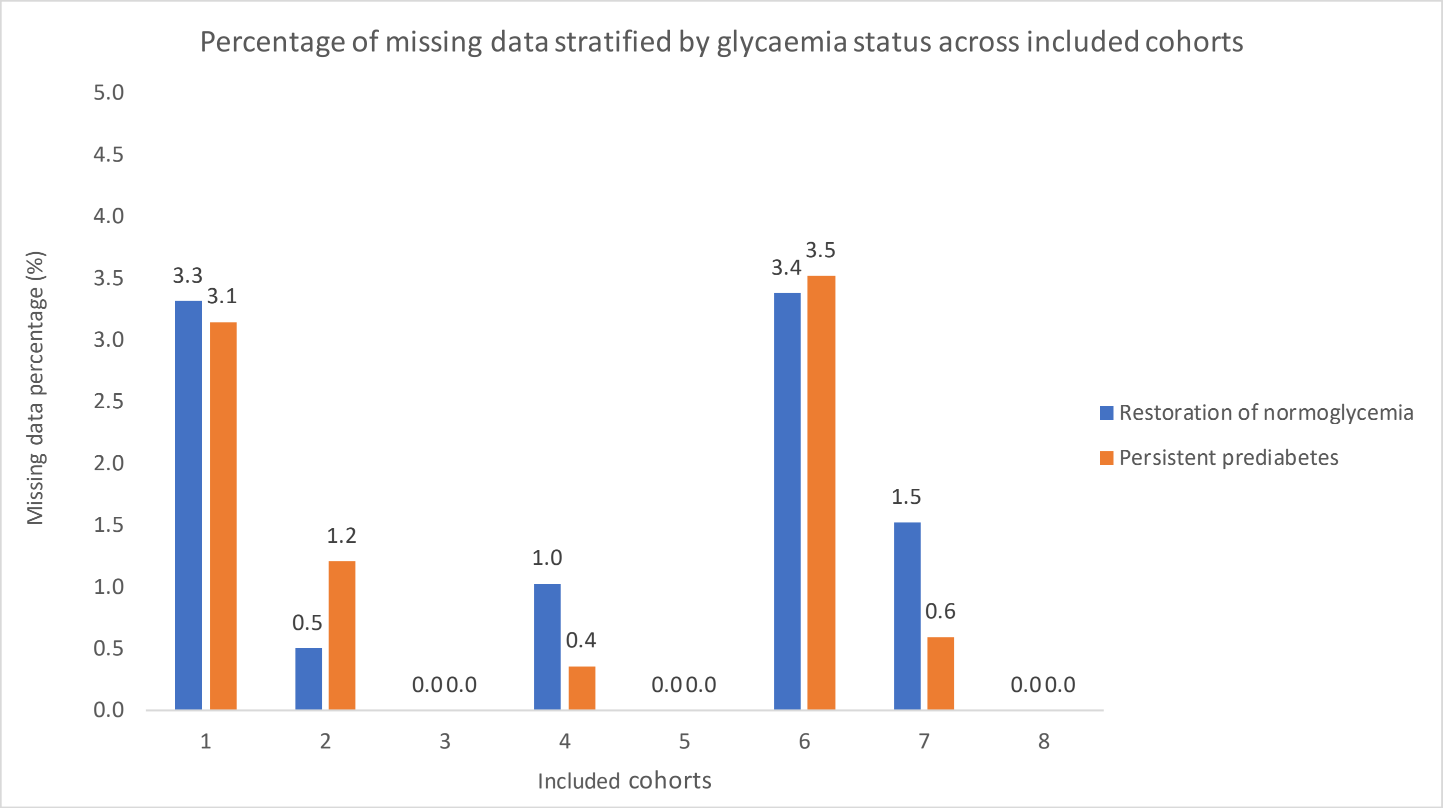

ESM Fig. 3. Missing data percentages across included cohorts, stratified by glycaemic status.

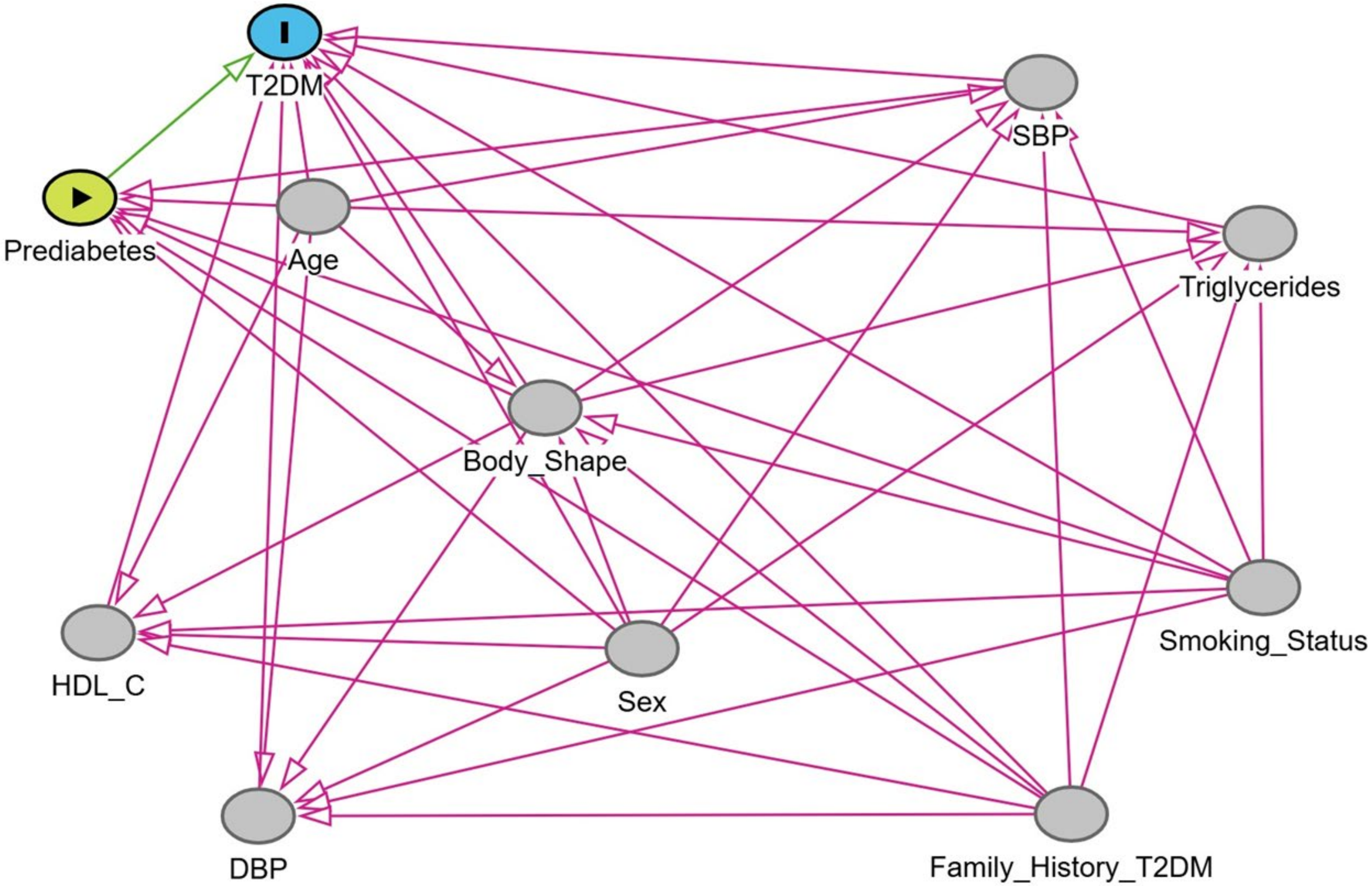

ESM Fig. 4. Directed acyclic graph illustrating hypothesized causal pathways between risk factors and prediabetes progression to type 2 diabetes.

This directed acyclic graph (DAG) illustrates the hypothesized causal relationships among prediabetes, regression to normoglycaemia, and the risk of type 2 diabetes, grounded in evidence from the literature. Cardiometabolic risk factors, such as body shape, are hypothesized to act as mediators on the causal pathway between regression to normoglycaemia and type 2 diabetes by influencing insulin sensitivity and β-cell function, key mechanisms in glucose regulation. Smoking status, systolic and diastolic blood pressure (SBP and DBP), Triacylglycerols, and HDL-C are included as potential confounders or effect modifiers, given their established roles in influencing the risk of type 2 diabetes. Family history of type 2 diabetes is representing genetic predisposition, while age and gender are included to account for demographic variations and their potential confounding effects.

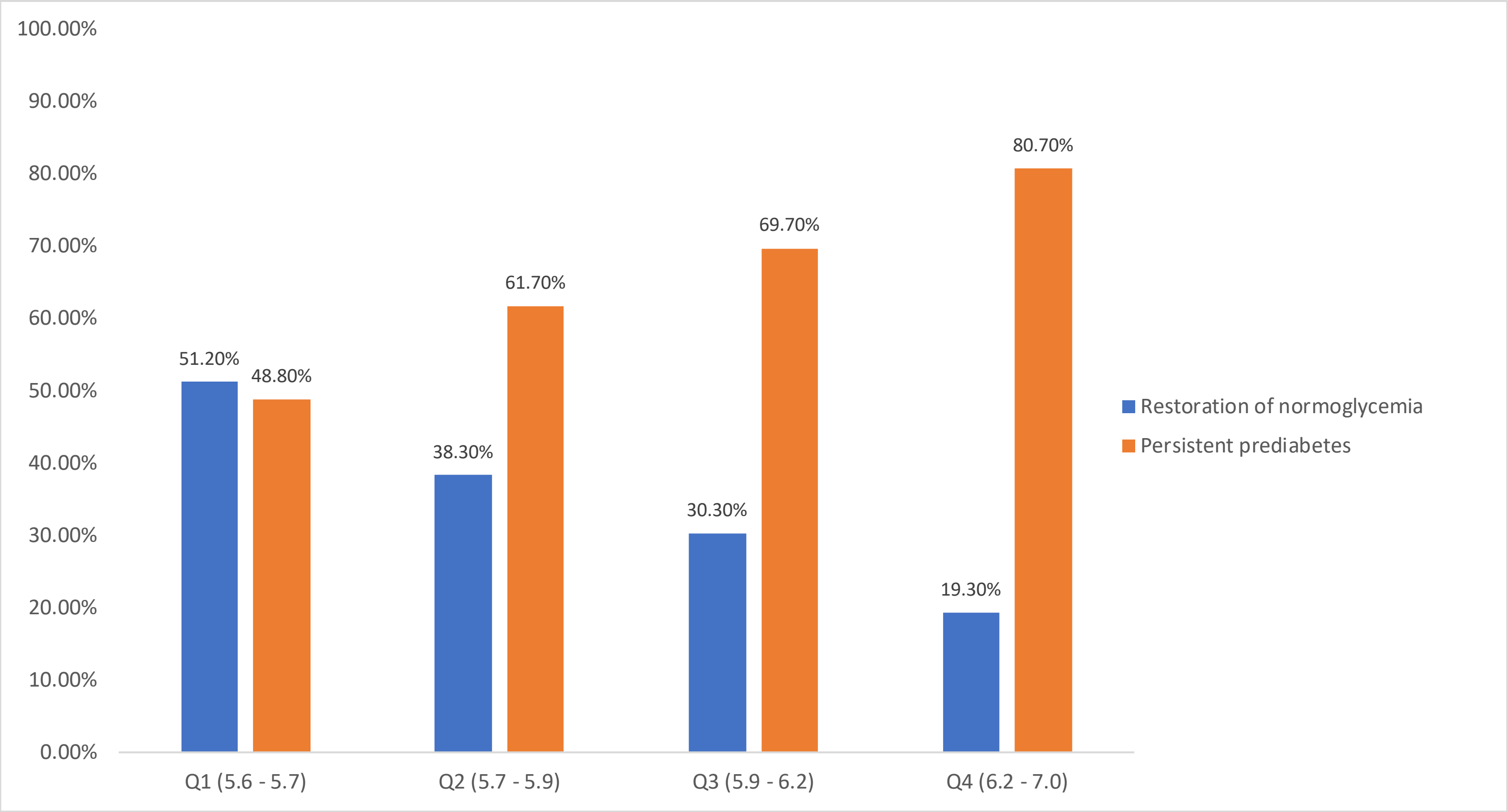

ESM Fig. 5. Percentage of participants who restored normoglycaemia or remained in the prediabetes stage at first follow-up, by quartile of fasting plasma glucose at baseline.

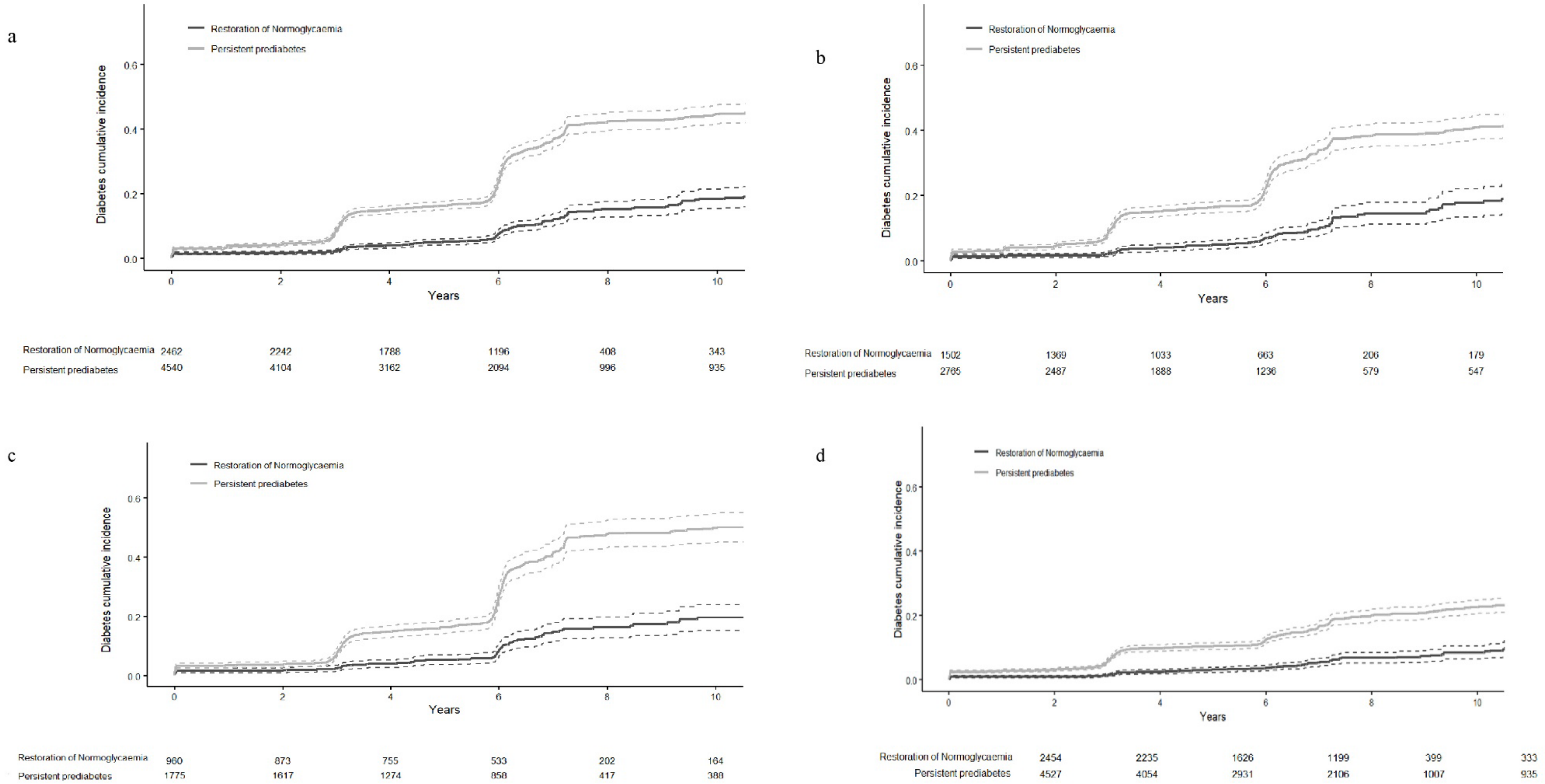

ESM Fig. 6. Nelson-Aalen plot of age-adjusted total and gender-specific cumulative risk of type 2 diabetes in individuals with prediabetes, stratified by glucose status at the first follow-up into those who transitioned to normoglycaemia and those with persistent prediabetes.

Total population (a), Male participants (b), Female participants (c), Sensitivity analysis (defining type 2 diabetes based on two abnormal FPGs) (d). Dashed lines show 95% CIs. *p<0·0001 between groups

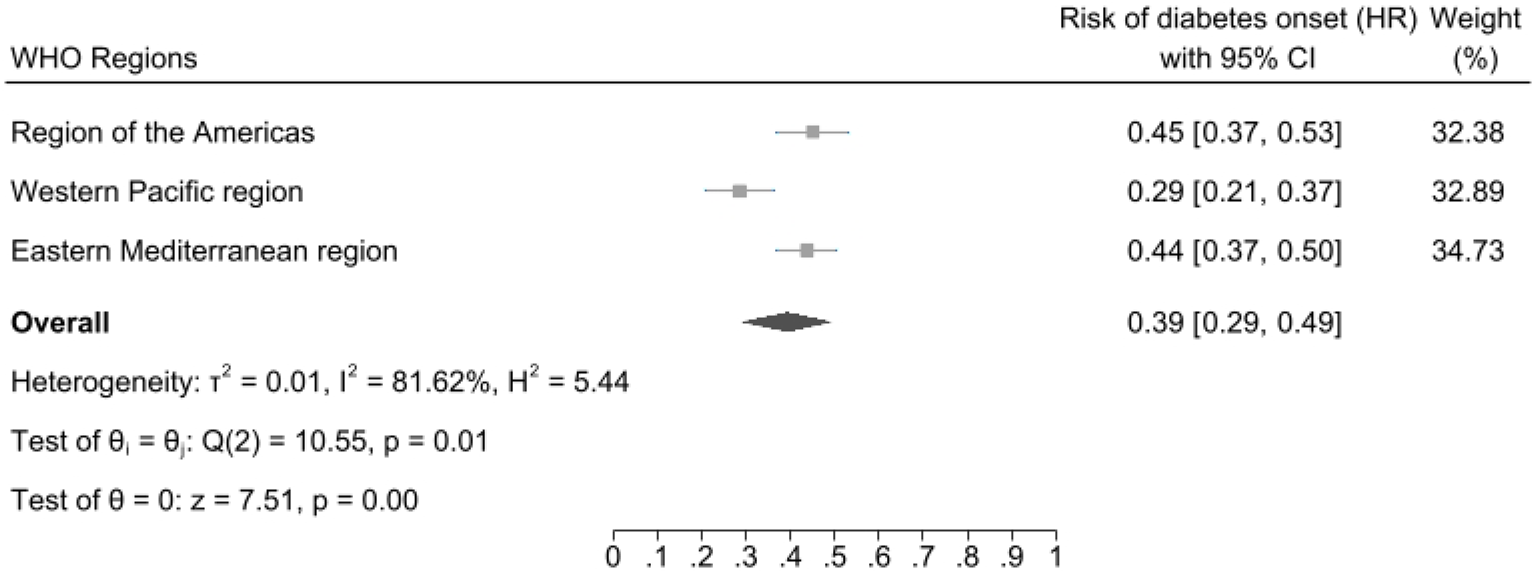

ESM Fig. 7. Hazard Ratios for diabetes onset comparing individuals who restored normoglycaemia to those with persistent prediabetes across WHO Regions

Compared group	Reference group	HR	95% CI
Normal WHtR and normoglycaemia	Elevated WHtR and prediabetes	0.15	[0.03, 0.26]
Normal WHtR and normoglycaemia	Normal WHtR and prediabetes	0.25	[0.04, 0.45]
Elevated WHtR and normoglycaemia	Elevated WHtR and prediabetes	0.26	[0.17, 0.36]
Normal WHR and normoglycaemia	Elevated WHR and prediabetes	0.17	[0.05, 0.28]
Normal WHR and normoglycaemia	Normal WHR and prediabetes	0.22	[0.05, 0.40]
Elevated WHR and normoglycaemia	Elevated WHR and prediabetes	0.32	[0.18, 0.46]
Normal SBP and normoglycaemia	Elevated SBP and prediabetes	0.20	[0.11, 0.30]
Normal SBP and normoglycaemia	Normal SBP and prediabetes	0.25	[0.14, 0.37]
Elevated SBP and normoglycaemia	Elevated SBP and prediabetes	0.27	[0.15, 0.40]
Normal DBP and normoglycaemia	Elevated DBP and prediabetes	0.25	[0.12, 0.38]
Normal DBP and normoglycaemia	Normal DBP and prediabetes	0.29	[0.16, 0.41]
Elevated DBP and normoglycaemia	Elevated DBP and prediabetes	0.24	[0.12, 0.35]
Normal TG and normoglycaemia	Elevated TG and prediabetes	0.24	[0.13, 0.35]
Normal TG and normoglycaemia	Normal TG and prediabetes	0.25	[0.14, 0.35]
High TG and normoglycaemia	High TG and prediabetes	0.29	[0.15, 0.44]
Normal HDL-C and normoglycaemia	Low HDL-C and prediabetes	0.21	[0.13, 0.29]
Normal HDL-C and normoglycaemia	Normal HDL-C and prediabetes	0.44	[0.19, 0.69]
Low HDL-C and normoglycaemia	Low HDL-C and prediabetes	0.21	[0.13, 0.30]

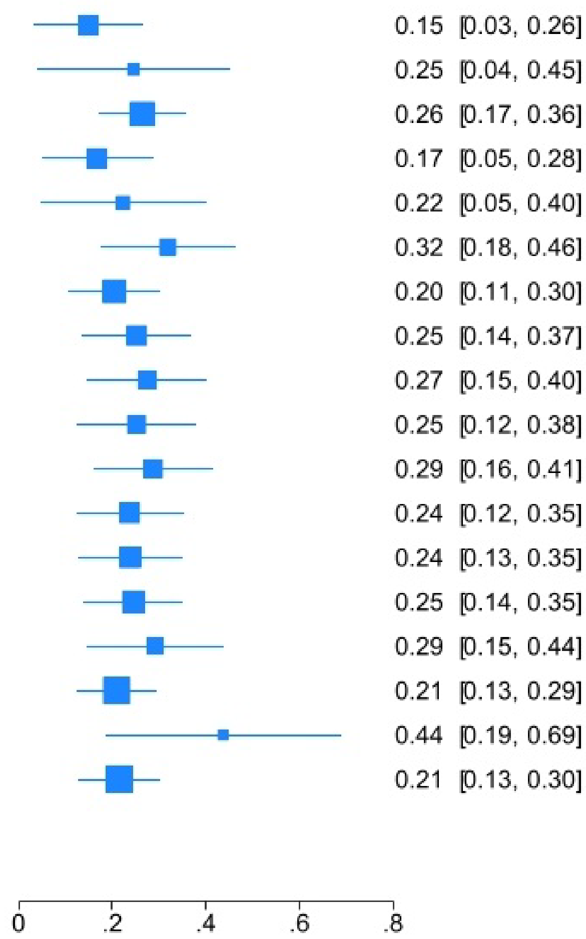

ESM Fig. 8. Model-adjusted hazard ratios (HRs) extracted from subgroup analyses estimating the simultaneous effect of restoring normoglycaemia and having normal cardiometabolic risk factor levels on the risk of type 2 diabetes.

The model is adjusted for age, gender, self-reported family history of type 2 diabetes in the first-degree relatives, smoking status, systolic-diastolic blood pressure, adiposity indices (BMI, WC, WHtR, WHR), serum HDL-C, and Triacylglycerols levels. To mitigate the effect of multicollinearity, the role of each adiposity index was explored in a separate model.
Square sizes represent the variability in sample sizes for each risk factor, as some studies did not measure all risk factors.
Abbreviations: BMI, body mass index; DBP, diastolic blood pressure; SBP, systolic blood pressure; TG, Triacylglycerols; HDL, high-density lipoprotein; FH, self-reported family history of type 2 diabetes in the first-degree relative; HR, Hazard ratio.

References

1. Uemura M, Yatsuya H, Hilawe EH, Li Y, Wang C, Chiang C, et al. Breakfast Skipping is Positively Associated With Incidence of Type 2 Diabetes Mellitus: Evidence From the Aichi Workers’ Cohort Study. Journal of Epidemiology. 2015;25(5):351-8.
2. investigators TA. The Atherosclerosis Risk in Communities (ARIC) Study: design and objectives. The ARIC investigators. Am J Epidemiol. 1989;129(4):687-702.
3. Center BSaDRIC. BioLINCC [Available from: https://biolincc.nhlbi.nih.gov/studies/aric/.
4. Dunstan DW, Zimmet PZ, Welborn TA, Cameron AJ, Shaw J, de Courten M, et al. The Australian Diabetes, Obesity and Lifestyle Study (AusDiab)—methods and response rates. Diabetes Research and Clinical Practice. 2002;57(2):119-29.
5. Taylor HA, Jr., Wilson JG, Jones DW, Sarpong DF, Srinivasan A, Garrison RJ, et al. Toward resolution of cardiovascular health disparities in African Americans: design and methods of the Jackson Heart Study. Ethn Dis. 2005;15(4 Suppl 6):S6-4-17.
6. Bild DE, Bluemke DA, Burke GL, Detrano R, Diez Roux AV, Folsom AR, et al. Multi-Ethnic Study of Atherosclerosis: Objectives and Design. American Journal of Epidemiology. 2002;156(9):871-81.
7. Grant JF, Taylor AW, Ruffin RE, Wilson DH, Phillips PJ, Adams RJ, et al. Cohort Profile: The North West Adelaide Health Study (NWAHS). Int J Epidemiol. 2009;38(6):1479-86.
8. Azizi F, Rahmani M, Emami H, Mirmiran P, Hajipour R, Madjid M, et al. Cardiovascular risk factors in an Iranian urban population: Tehran Lipid and Glucose Study (Phase 1). Sozial- und Präventivmedizin. 2002;47(6):408-26.
9. Sun Y, Sekine M, Kagamimori S. Lifestyle and overweight among Japanese adolescents: the Toyama Birth Cohort Study. Journal of epidemiology. 2009;19(6):303-10.
10. Alberti KG, Zimmet P, Shaw J. The metabolic syndrome--a new worldwide definition. Lancet. 2005;366(9491):1059-62.
11. Alberti KGMM, Zimmet P, Shaw J. International Diabetes Federation: a consensus on Type 2 diabetes prevention. Diabetic Medicine. 2007;24(5):451-63.
12. Azizi F, Khalili D, Aghajani H, Esteghamati A, Hosseinpanah F, Delavari A, et al. Appropriate waist circumference cut-off points among Iranian adults: the first report of the Iranian National Committee of Obesity. Arch Iran Med. 2010;13(3):243-4.
